# The Relationship Between Weight Loss Outcomes and Engagement in a Mobile Behavioral Change Intervention: Retrospective Analysis

**DOI:** 10.2196/30622

**Published:** 2021-11-08

**Authors:** Alissa Carey, Qiuchen Yang, Laura DeLuca, Tatiana Toro-Ramos, Youngin Kim, Andreas Michaelides

**Affiliations:** 1 Academic Research Noom Inc New York, NY United States; 2 Department of Clinical Psychology Ferkauf Graduate School of Psychology Yeshiva University Bronx, NY United States; 3 Amgen Thousand Oaks, CA United States; 4 Department of Biomedical Systems Informatics Yonsei University College of Medicine Seoul Republic of Korea

**Keywords:** engagement, mHealth, obesity, weight management, Noom, application, app, behavioral change, digital behavior change interventions

## Abstract

**Background:**

There is large variance in weight loss outcomes of digital behavior change interventions (DBCIs). It has been suggested that different patterns of engagement in the program could be responsible for this variance in outcomes. Previous studies have found that the amount of engagement on DBCIs, such as the number of meals logged or articles read, is positively associated with weight loss.

**Objective:**

This retrospective study extends previous research by observing how important weight loss outcomes (high weight loss: 10% or greater body weight loss; moderate weight loss: between 5% to 10%; stable weight: 0 plus or minus 1%) are associated with engagement on a publicly available mobile DBCI (Noom) from 9 to 52 weeks.

**Methods:**

Engagement and weight data for eligible participants (N=11,252) were extracted from the Noom database. Engagement measures included the number of articles read, meals logged, steps recorded, messages to coach, exercise logged, weigh-ins, and days with 1 meal logged per week. Weight was self-reported on the program. Multiple linear regressions examined how weight loss outcome (moderate and high vs stable) was associated with each engagement measure across 3 study time periods: 9-16 weeks, 17-32 weeks, and 33-52 weeks.

**Results:**

At 9-16 weeks, among the 11,252 participants, 2594 (23.05%) had stable weight, 6440 (57.23%) had moderate weight loss, and 2218 (19.71%) had high weight loss. By 33-52 weeks, 525 (18.21%) had stable weight, 1214 (42.11%) had moderate weight loss, and 1144 (39.68%) had high weight loss. Regression results showed that moderate weight loss and high weight loss outcomes were associated with all engagement measures to a significantly greater degree than was stable weight (all *P* values <.001). These differences held across all time periods with the exception of exercise for the moderate weight loss category at 1 time period of 33-52 weeks. Exercise logging increased from 9 to 52 weeks regardless of the weight loss group.

**Conclusions:**

Our results suggest that these clinically important weight loss outcomes are related to the number of articles read, meals logged, steps recorded, messages to coach, exercise logged, weigh-ins, and days with 1 meal logged per week both in the short-term and long-term (ie, 1 year) on Noom. This provides valuable data on engagement patterns over time on a self-directed mobile DBCI, can help inform how interventions tailor recommendations for engagement depending on how much weight individuals have lost, and raises important questions for future research on engagement in DBCIs.

## Introduction

### Engagement and Digital Behavior Change Interventions

Obesity and its potential comorbidities are a significant and increasing public health burden, with an estimated global cost of US $2 trillion per year due to economic loss of productivity and direct medical expenses stemming from weight-related issues [1]. Traditional dietary approaches to treat obese and overweight status have known shortcomings, calling for innovative solutions that involve behavioral management [2,3]. Digital behavior change interventions (DBCIs) such as mobile programs use technology to enhance availability and convenience compared to traditional in-person interventions, and these programs are growing in number [4-6]. These digital interventions are effective for weight loss and chronic disease prevention and management [7-10].

Body weight loss of 5%-10% is associated with improved risk of metabolic and cardiovascular conditions, and 10% or more loss is associated with even greater improvement [11]. Therefore, body weight loss of at least 5% is regarded as a clinically meaningful outcome [12]. However, there is wide variability in weight loss outcomes even when individuals use the same program [13,14]. It has been suggested that this variability could be due to differences in engagement with the program [15]. Engagement has been defined as “the extent (eg, amount, frequency, duration, depth) of usage” of the program [16]. Common measures of engagement include the amount of time spent on the platform, the number of times an individual has used a program feature such as weight or food logging, and the number of articles read [15].

### Previous Work on Engagement

Previous work has found positive associations between engagement and weight loss outcomes [10,17-22]. In a digital commercial program, the number of weigh-ins per week, steps per day, active minutes per week, days logging meals per week, and the percentage of weeks with 5 or more meal logs were associated with weight loss at 6 months [19]. In the same study, weighing in at least 3 times a week, achieving 60 highly active minutes per week, and logging meals 3 times per week were associated with 5% or more body weight loss [19]. In our previous work on Noom, a commercial mobile DBCI, we found that the number of meal logs and group posts were associated with greater weight loss at 65 weeks, and the number of messages sent to the coach, exercises logged, and articles read were associated with weight gain [20]. We also previously found that the number of meal logs and weigh-ins were associated with weight loss at 6 months on Noom [10].

### This Study

We extend this body of work in this retrospective study by examining how specific weight loss outcomes of interest are associated with engagement using a large sample and multiple time points. Multiple time points allow for the investigation of whether associations change over time, which is important because it is well established that engagement declines over time [23]. This study will allow for better understanding of how individuals who lost certain amounts of weight engaged in the program, which can inform future attempts to encourage engagement in specific and tailored ways based on current weight and goal weight loss. Specifically, we explored associations between weight loss outcomes of clinical importance (5%-10%, 10% and more, and stable weight) and various measures of engagement (the number of articles read, meals logged, steps recorded, messages to coach, exercise logged, weigh-ins, and days with 1 meal logged per week) from 9 to 52 weeks on Noom. Based on past work, it was hypothesized that associations between weight loss outcomes and the number of meal logs and weigh-ins would be stronger for moderate (5%-10%) or higher amounts of weight loss (10% or more) compared to stable weight loss outcomes [10,19,20], but it was unclear if that would be the case for all engagement measures because of mixed prior results [18]. We also hypothesized that the difference in engagement between these weight loss groups would hold over time [18].

## Methods

### Intervention

Noom is a behavior change and weight management mobile health intervention that provides users with self-monitoring features for food, exercise, and weight monitoring, as well as access to a virtual 1:1 behavior change coach, support group facilitated by a health coach, and a daily curriculum that includes diet, exercise, and psychoeducation. Noom’s theoretical foundation stems from cognitive behavioral therapy; third wave cognitive behavioral therapy, such as dialectical behavioral therapy and motivational interviewing techniques; and behavior change techniques, such as self-monitoring and social support [24-27].

### Participants

Retrospective cohort data were extracted directly from Noom’s (Noom Inc) database in December 2019 and deidentified. Participants had all voluntarily signed up for the Noom Healthy Weight program online or through the app store (iTunes or Google Play). This study was approved by the Advarra Inc Institutional Review Board (Columbia, Maryland). As part of the approved protocol, at initial sign-up, all users were given an opportunity to consent to the use of all of their program data for research, and all users were given the opportunity to opt out and deny consent.

To be included in this study, individuals were required to be Noom users in the Healthy Weight program for up to 52 weeks beginning on December 1, 2018; had provided baseline weight, age, gender, and height; were 18 years or older; and fell into one of the 3 weight loss outcome categories used in the study. A length of 52 weeks was chosen to be able to explore long-term weight loss and engagement [28]. Additionally, participants had to open the mobile health platform at least once after week 8 to be included in the study as a minimum threshold of activity. Week 8 was chosen because this would represent activity from week 9 onwards, which is when the first study time period began.

All participants were placed into 1 of 3 weight outcome categories based on their weight change from baseline: stable weight (0% plus or minus 1%), moderate weight loss (between 5% and 10%), and high weight loss (lost 10% or more body weight). These categories represent clinically meaningful weight loss outcomes, and labels were chosen following previous work [29,30].

The following time periods were chosen for analysis: 9-16 weeks, 17-32 weeks, and 33-52 weeks. The initial time period was chosen based on program length (16 weeks), and the starting point was set to halfway through the program (9 weeks) to prevent bias from early fluctuations in motivation or weight. The final time period (52 weeks) was designated based on previous work [31], and the middle time period (32 weeks) was chosen to represent an intermediate interval between the initial and final time periods. To be included at later time periods, participants had to fall into 1 weight change category and have opened the platform at least once during week 16 to be analyzed at weeks 17 to 32, and have opened the platform at least once in week 32 in order to be analyzed in weeks 33 to 52.

### Measures

Weight, as well as baseline characteristics of gender, age, and height were self-reported by the users through the mobile interface.

The following engagement measures were used: number of days with at least 1 meal logged per week—a measure calculated based on participants’ weekly self-reported food logs; number of articles read per week; number of meal logs per week—the number of meals participants logged in the platform per week; number of coach messages per week—the number of times participants messaged their coach per week; count of steps per week—the number of steps taken per week, either recorded by the participant’s in-phone pedometer or supplemented by self-report in the platform; count of weigh-ins per week—the number of times participants self-reported their weight in the platform per week; and count of exercises per week—the number of times participants self-reported exercising in the platform per week. These engagement measures comprehensively included the possible ways users could actively participate on the platform.

No engagement measures were required as part of the intervention. The curriculum content (articles) functioned on a fixed schedule where participants were shown potential articles to read containing nutrition education, psychoeducation, and motivational information each day. They were encouraged at the beginning of the program to read these articles as part of a daily task list. Participants were also encouraged to perform weight logging at least once a week and to log all of their meals daily. Participants had the option of setting up push notifications to remind them to log their meals at certain times. Using this optional reminder system was not tracked as an engagement measure. Coaches were instructed to reply to user messages within 24 hours of receiving them, and, if the participant did not send the coach a message in 7 days, the coach would reach out with a weekly check-in to invite discussion over the participant’s progress. Participants’ engagement with coach messaging was calculated based on messages that they sent, not messages received.

### Statistical Analysis

Descriptive statistics were calculated for users’ baseline characteristics and are expressed in mean and SD for continuous variables and frequency and percentage for categorical variables (Table 1). These characteristics were self-reported, including users’ weights, which were measured by the users with their own scales.

**Table 1:** Demographic characteristics for weight change groups across study periods.

Characteristic		Weight change groups			*P* value
		Stable (0 plus or minus1%)	Moderate loss (between 5%-10%)	High loss (10% or greater)	
**Age (years), mean (SD)**					
	9-16 weeks	47.70 (12.27)	49.93 (12.59)	49.67 (12.4)	<.001
	17-32 weeks	47.89 (12.16)	50.37 (12.51)	50.51 (12.33)	<.001
	33-52 weeks	49.45 (11.71)	51.57 (12.32)	51.8 (12.18)	<.001
**Gender, n/N (%)**					
	9-16 weeks	235/2594 (9.1)	738/6440 (11.5)	347/2218 (15.6)	<.001
	17-32 weeks	166/1907 (8.7)	447/4369 (10.2)	356/2686 (13.2)	<.001
	33-52 weeks	67/525 (12.8)	131/1214 (10.8)	126/1144 (11.0)	.47
**Baseline BMI, mean (SD)**					
	9-16 weeks	26.80 (5.58)	26.57 (5.5)	27.19 (5.24)	<.001
	17-32 weeks	26.97 (5.78)	26.73 (5.52)	27.43 (5.45)	<.001
	33-52 weeks	27.10 (5.8)	27.38 (5.34)	28.11 (5.43)	.001

#### Overall Engagement by Weight Loss Group 

Multivariate analysis of variance (MANOVA) was used to examine if overall engagement significantly differed across the 3 weight loss groups for each time period. Assumptions for MANOVA were checked and met.

#### Individual Engagement Measures by Weight Loss Group

Multiple linear regressions predicted the engagement associated with each weight outcome of interest. Regressions were conducted with overall mean weekly engagement (per time period) of each engagement measure as separate dependent variables in individual regression analyses and the weight loss category as the independent variable, with controlling for baseline characteristics of age, gender, and BMI. Self-reported engagement data were excluded from the overall mean calculation if missing data were found in any week during each time period, as it would unclear if the missing data indicated a lack of engagement or a lack of reporting. For automatically recorded measures (eg, steps), any missingness in data during a time point was kept in the total mean calculation, as this is an indicator of lack of engagement. Assumptions for linear regression were checked and met.

All statistical tests were 2-sided with significance set at a *P* value <.05 and were conducted through R version 3.6.0 (The R Foundation for Statistical Computing).

## Results

### Baseline Characteristics

Of the 11,252 participants observed at 9-16 weeks, 23.05% (n=2594) of participants were in the stable weight category, 57.23% (n=6440) were in the moderate weight loss category, and 19.71% (n=2218) were in the high weight loss category. Of the 8962 participants observed at weeks 17-32, 21.28% (n=1907) were in the stable weight category, with 48.75% (n=4369) in the moderate weight loss category and 29.97% (n=2686) in the high weight loss category. Finally, by 33-52 weeks, 18.21% (525/2883) were in the stable weight category, with 42.11% (1214/2883) in the moderate weight loss category and 39.68% (1144/2883) in the high weight loss category.

Of the participants observed at weeks 9-16, 88.45% (9952/11,252) were female, with a mean age of 49.43 (SD 12.54) and a mean BMI of 26.75 (SD 5.47). At weeks 17-32 and 33-52, the majority of users observed were female (17-32 weeks: 10036/11,252, 89.19%; 33-52 weeks: 9987/11,252, 88.76%), with a mean age of 49.93 (SD 12.46) during weeks 17-32 and a mean age of 51.24 (SD 12.20) during weeks 33-52. During weeks 9-16, the mean baseline BMI was 26.8 (SD 5.57) kg/m^2^ for the stable group, 26.57 (SD 5.50) kg/m^2^ for the moderate loss group, and 27.19 (SD 5.24) kg/m^2^ for the high loss group. These baseline characteristics are included in Table 1.

Significant differences existed between the stable, moderate, and high weight loss groups during weeks 9-16 regarding age (*F*_2,11249_=30.08; *P*<.001), gender (χ^2^_2_=50.78; *P*<.001), and BMI (*F*_2,11249_=10.54; *P*<.001); and during weeks 17-32, age (*F*_2,8959_ = 29.06; *P*<.001), gender (χ^2^_2_=30.69; *P*<.001), and BMI (*F*_2,8959_ = 13.73; *P*<.001). These differences remained significant during weeks 32-52 for BMI (*F*_2,2880_ = 8.38; *P*<.001) and age (*F*_2,2880_ = 7.37; *P*<.001), but not for gender (χ^2^_2_ = 1.39; *P*=.50). As a result, we adjusted these demographic measures in the regression analysis. These overall means are described in Table 1.

**Overall Engagement by Weight Loss Group**

The multivariate analysis of variance test resulted in statistically significant differences in overall engagement among the 3 weight categories for weeks 9-16 (*F*_2,11249_ = 197.43; *P*<.001), for weeks 17-32 (*F*_2,8959_=153.50; *P*<.001), and for weeks 33-52 (*F*_2,2880_ = 44.26; *P*<.001). Therefore, we concluded that for each study time period, engagement as a whole, consisting of mean days with at least 1 meal logged per week, mean articles read per week, mean meals logged per week, mean user messages, mean steps, mean weigh-ins per week, and mean exercises logged per week, significantly differed across the 3 weight categories. Engagement as a whole was highest for the high weight loss group, followed by the moderate weight loss group, with the lowest engagement in the stable group. The results of the MANOVA can be seen in Table S1 in Multimedia Appendix 1.

**Individual Engagement Measures by Weight Loss Group**

When examined individually, means of the following engagement measures decreased over time across all weight groups: days with at least 1 meal logged, articles read, number of meals logged, steps, coach messages, and weigh in variables. Logged exercise did not follow the same pattern, as total mean logged exercise increased through 52 weeks, regardless of the total amount of weight lost (see Table S2 in Multimedia Appendix 1).

The patterns of means suggest that the moderate and high weight loss groups had greater total engagement within each study time period across all engagement measures compared to the stable weight group. To confirm that differences between the weight loss groups and the stable group were significant and to examine the relationship between weight loss outcomes and each engagement measure, individual multiple regressions were conducted with the stable group as the reference group (see Table 2). Differences in engagement between the high weight loss group and moderate loss group were not examined due to a lack of a clinically meaningful difference between these 2 groups given that significant health improvements occur when weight loss exceeds 5% [12,29]. Therefore, we focused our statistical analysis on comparing the high loss group to the stable group and the moderate loss group to the stable group.

**Table 2:** Multiple regression results for weight change groups in each study period^a^.

Engagement measures^b^			Time points																	
			9-16 weeks						17-32 weeks							33-52 weeks				
			Estimate		SE		Adj^c^ *R*^2^		Estimate		SE		Adj *R*^2^		Estimate			SE		Adj *R*^2^
**Days with at least 1 meal logged**																				
	Moderate loss	0.20		0.01		N/A^d^		0.25		0.01		N/A		0.24			0.02		N/A	
	High loss	0.25		0.01		0.05		0.34		0.01		0.08		0.37			0.02		0.12	
**Articles read**																				
	Moderate loss	0.52		0.03		N/A		0.89		0.05		N/A		0.63			0.07		N/A	
	High loss	0.68		0.04		0.05		1.20		0.05		0.06		1.01			0.07		0.05	
**Meals logged**																				
	Moderate loss	5.83		0.15		N/A		5.25		0.19		N/A		4.58			0.37		N/A	
	High loss	8.06		0.19		0.16		8.27		0.21		0.20		8.22			0.37		0.17	
**Coach messages**																				
	Moderate loss	0.06		0.01		N/A		0.07		0.01		N/A		0.08			0.01		N/A	
	High loss	0.09		0.01		0.01		0.10		0.01		0.01		0.12			0.01		0.03	
**Steps**																				
	Moderate loss	4014.04		483.60		N/A		4452.65		579.76		N/A		4535.31			1165.38		N/A	
	High loss	8806.42		601.71		0.05		9821.59		633.01		0.06		8963.01			1176.49		0.05	
**Weigh ins**																				
	Moderate loss	0.23		0.01		N/A		0.26		0.01		N/A		0.16			0.017		N/A	
	High loss	0.29		0.01		0.17		0.34		0.01		0.16		0.27			0.017		0.10	
**Exercises**																				
	Moderate loss	0.25		0.07		N/A		0.28		0.08		N/A		0.17			0.16		N/A	
	High loss	0.51		0.09		0.01		0.77		0.09		0.01		0.87			0.16		0.02	

^a^The stable group was used as the reference group.

^b^Results are the summary of 7 individual multiple linear regressions. Each engagement measure was a dependent variable of its own regression where weight change groups, gender, age and baseline BMI were independent variables.

^c^Adj: adjusted.

^d^N/A: not applicable.

When users’ age, gender, and baseline BMI were controlled for, the moderate and high weight loss groups had significantly more days with at least 1 meal logged (*P* values <.001), articles read (*P* values <.001), meals logged (*P* values <.001), coach messages (*P* values <.001), steps (*P* values <.001), and weigh ins (*P* values <.001) compared to the stable group across all time points. The moderate and high weight loss groups had greater mean exercise per week compared to the stable weight group at 9-16 weeks and 17-32 weeks (*P* values <.001). For weeks 33-52, only the high loss group had significantly greater mean frequency of exercise compared to the stable group (*β*=.87; SE=0.16; *P*<.001).

## Discussion

### Principal Results

Using a large data set of more than 11,000 individuals, we sought to extend previous work by evaluating how specific weight loss outcomes (stable weight, 0% plus or minus 1%; moderate weight loss, between 5% to 10%; and high weight loss, 10% or greater) were associated with 7 different engagement measures across 3 time periods: 9-16 weeks, 17-32 weeks, and 33-52 weeks. Overall, our findings indicate significant differences in all 7 engagement measures among those with moderate and high weight loss compared to those with stable weight. These associations held over time, with the exception of exercise logging at 33-52 weeks for the moderate weight loss category.

### Comparison With Prior Work

Our findings corroborate past work that found significant associations between the frequency of food logging and weigh-ins and weight loss outcomes [10,17-22]. Departing from some past studies, we found that weight loss outcomes were associated with all engagement measures over all time periods, with one exception. In contrast, some previous work has reported significant associations between engagement and weight loss for some, but not all, engagement measures [18,21]. This discrepancy may be due to the fact that previous studies explored the amount of engagement necessary to achieve a certain amount of weight loss (ie, how engagement is associated with weight loss), whereas our study is concerned with understanding how achieving a certain level of weight loss is related to levels of engagement (ie, how weight loss is associated with engagement). Our findings raise the possibility that individuals who achieve successful weight outcomes tend to engage comprehensively in the program because they are generally more motivated, whereas only certain engagement measures are necessary to achieve greater weight loss. Future research should explore within-participant patterns of engagement across time as related to individual differences such as motivation or personality characteristics.

According to recent conceptual models of engagement, the content of the intervention (eg, availability of self-monitoring tools), contextual factors, and psychological characteristics like motivation and self-efficacy can influence engagement [16,32]. This study raises additional questions about weight loss outcomes, which could also influence engagement. It is possible that the factors driving individuals to successfully achieve certain levels of weight loss influence their engagement as well. We previously demonstrated that 5% or more weight loss on Noom was associated with psychosocial characteristics such as mental health quality of life and perceived work-life balance [33], and a systematic review reported that weight loss is associated with the expectations individuals have for their weight loss [14]. Future research should separate out the individual components involved in losing certain amounts of weight loss and investigate how each relates to engagement.

Through the large sample size and year-long time period, this study provides data of how engagement decreases over time. In general, the levels of engagement decreased over time for 6 of the 7 engagement measures (with the exception of exercise logging) within each group regardless of whether they achieved no loss, moderate loss, or high loss. This is consistent with past work showing declines in some engagement measures over time but not for physical activity logging [34]. Future research should investigate the possibility that sustained exercise habits are formed on this type of program. We also found that the differences between each weight loss category and stable weight were maintained even as engagement decreased over time. This aligns with a study showing that associations between engagement and weight change were consistent from 16 weeks through 52 weeks [18]. It is necessary for future research to test whether these results mean that long-term engagement accurately reveals true patterns of motivation and action, or whether late engagement is instead a marker of “user’s behavior chang[ing] to an extent that digital engagement with the intervention is no longer needed” [35], or both.

Along these lines, we found that being in the moderate weight loss group at 33-52 weeks was not associated with the frequency of exercise logging. There are a few possible reasons for this. Perhaps as they were initially losing weight, individuals in the moderate group perceived that their modest weight loss was primarily due to dietary change rather than exercise, and then they were less likely to consistently log exercise. A previous survey study found that 71% of respondents assumed that exercise is an effective weight loss strategy, and this assumption was associated with feeling discouraged with exercise [36]. Alternatively, perhaps moderate weight loss is more associated with types of logging that require greater effort, such as meal logging and weight logging that users are encouraged to do daily, in contrast to exercise logging which only occurs after individuals have exercised. In our results, the weight loss category accounted for more of the variation in meal logging (adjusted *R*^2^=0.16) and weight logging (adjusted *R*^2^=0.17) than did other engagement measures (adjusted *R*^2^=0.01-0.05). Similarly, in a previous study, meal logging and weight logging predicted changes in weight more than did exercise logging [37]. Future studies should investigate why and how meal and weight logging may differ from exercise logging.

### Limitations

The study’s strengths include exploring real-world engagement in a large sample on a publicly available mobile DBCI. Some limitations, however, should be noted. First, a convenience sample of individuals who had self-selected to sign up for the Noom Healthy Weight program was used. Thus, findings may not generalize to populations with less motivation to manage their weight. Given the retrospective design, causal relationships between participants’ engagement measures and participants’ weight loss outcomes cannot be determined. The correlational nature of analyses also prevents firm conclusions about the directionality of results. Finally, the users measured and reported their own weight in the platform, and their loss was calculated from these self-reported measures. Home scales may produce a considerable margin of error compared to ones used by health care professionals. Users were encouraged to use the same scale throughout the program so that their personal loss would be consistent with their individual scale. They were also encouraged to weigh in at the same time every day (ideally in the morning upon first waking up), but it was not possible to enforce these recommendations.

Weight loss was calculated based on self-reported weight measurements by participants, and baseline weight information was contextualized using BMI. Both measures are limited in a few ways. First, they do not adjust for the weight fluctuation that occurs during menstrual cycles or perimenopause and menopause. They also do not account for muscle mass or bone density. Individuals may have increased their muscle mass due to exercise, but this would not be adequately captured by these metrics. Finally, these measures could be subject to artificial inflation because of water retention due to excessive salt intake. Future studies should use a variety of self-reported and objective measurements to understand individuals’ weight changes.

### Conclusions

This retrospective study explored associations between important weight loss outcomes and engagement in a mobile DBCI over 1 year, which could help to inform tailoring interventions to encourage engagement based on achieved and goal weight loss outcomes or provide data that can be used to better understand variance in weight loss outcomes. This study also provides large-scale data on how individuals engage in a self-directed mobile DBCI in the short- and long-term. We found that compared to stable weight, having achieved moderate weight loss or high weight loss was associated with higher engagement in the forms of the number of meals logged, articles read, steps logged, coach messages, weigh-ins, and days with at least 1 meal logged from 9 to 52 weeks. This raises the possibility that individuals who lose moderate or high amounts of weight actively engage in all possible aspects of the program, which future research should confirm. The one exception was that being in the moderate weight loss category at 33-52 weeks was more associated with exercise logging than was being in the stable weight category. The consistent associations over time suggests that these differences in engagement behavior are stable throughout both short-term and long-term weight loss. Future research can ascertain to what extent our results are generalizable to other intervention contexts.

Our results raise new questions for future studies which should seek to more fully understand the engagement of individuals who lose significant weight. In this study, participants who achieved moderate weight loss, on average at 17-32 weeks, logged 21 meals, read 3 articles, walked 31,600 steps, weighed in once, exercised 2.5 times within a week, and messaged their coach once every 2 weeks. Users who achieved high weight loss on average logged 24 meals, read 3.4 articles, walked 36,000 steps, weighed in once, exercised 3 times within a week, and messaged their coach once every 2 weeks. These overall means do not take into account variation within users, but future work should go further to, for instance, define profiles of engagement based on weight loss outcomes. This could provide insight into the large variance in weight loss outcomes observed in many interventions.

Future work can explore the following questions: what exactly is responsible for the association between certain weight loss outcomes and engagement in a weight loss program? Is it seeing results, personality factors, demographic factors, or past success or failure with weight loss, or some combination of these? Would telling someone that x level of engagement is related to x level of loss be enough to change their behavior to better engage the mobile health intervention? The next steps may involve exploring other individual or contextual factors in order to understand what guides the engagement seen on this DBCI.

## Appendix

Multimedia Appendix 1 Supplementary Tables S1 and S2.

## Abbreviations

DBCI: digital behavior change intervention
MANOVA: multivariate analysis of variance

## References

[1] Swinburn Boyd A, Kraak Vivica I, Allender Steven, Atkins Vincent J, Baker Phillip I, Bogard Jessica R, Brinsden Hannah, Calvillo Alejandro, De Schutter Olivier, Devarajan Raji, Ezzati Majid, Friel Sharon, Goenka Shifalika, Hammond Ross A, Hastings Gerard, Hawkes Corinna, Herrero Mario, Hovmand Peter S, Howden Mark, Jaacks Lindsay M, Kapetanaki Ariadne B, Kasman Matt, Kuhnlein Harriet V, Kumanyika Shiriki K, Larijani Bagher, Lobstein Tim, Long Michael W, Matsudo Victor K R, Mills Susanna D H, Morgan Gareth, Morshed Alexandra, Nece Patricia M, Pan An, Patterson David W, Sacks Gary, Shekar Meera, Simmons Geoff L, Smit Warren, Tootee Ali, Vandevijvere Stefanie, Waterlander Wilma E, Wolfenden Luke, Dietz William H (2019). The global syndemic of obesity, undernutrition, and climate change: the Lancet Commission Report. Lancet.

[2] Dalcin AT, Jerome GJ, Fitzpatrick SL, Louis TA, Wang N, Bennett WL, Durkin N, Clark JM, Daumit GL, Appel LJ, Coughlin JW (2015). Perceived helpfulness of the individual components of a behavioural weight loss program: results from the Hopkins POWER Trial. Obes Sci Pract.

[3] Kopelman PG, Grace C (2004). New thoughts on managing obesity. Gut.

[4] Yardley L, Spring BJ, Riper H, Morrison LG, Crane DH, Curtis K, Merchant GC, Naughton F, Blandford A (2016). Understanding and promoting effective engagement with digital behavior change interventions. Am J Prev Med.

[5] Higgins JP (2016). Smartphone applications for patients' health and fitness. Am J Med.

[6] Elbert NJ, van Os-Medendorp H, van Renselaar RW, Ekeland AG, Hakkaart-van RL, Raat H, Nijsten TEC, Pasmans SGMA (2014). Effectiveness and cost-effectiveness of ehealth interventions in somatic diseases: a systematic review of systematic reviews and meta-analyses. J Med Internet Res.

[7] Hutchesson MJ, Rollo ME, Krukowski R, Ells L, Harvey J, Morgan PJ, Callister R, Plotnikoff R, Collins CE (2015). eHealth interventions for the prevention and treatment of overweight and obesity in adults: a systematic review with meta-analysis. Obes Rev.

[8] Little P, Stuart B, Hobbs FR, Kelly J, Smith ER, Bradbury KJ, Hughes S, Smith PWF, Moore MV, Lean MEJ, Margetts BM, Byrne CD, Griffin S, Davoudianfar M, Hooper J, Yao G, Zhu S, Raftery J, Yardley L (2016). An internet-based intervention with brief nurse support to manage obesity in primary care (POWeR+): a pragmatic, parallel-group, randomised controlled trial. Lancet Diabetes Endocrinol.

[9] Flores Mateo Gemma, Granado-Font E, Ferré-Grau Carme, Montaña-Carreras Xavier (2015). Mobile phone apps to promote weight loss and increase physical activity: a systematic review and meta-analysis. J Med Internet Res.

[10] Michaelides A, Raby C, Wood M, Farr K, Toro-Ramos T (2016). Weight loss efficacy of a novel mobile Diabetes Prevention Program delivery platform with human coaching. BMJ Open Diabetes Res Care.

[11] Ryan DH, Yockey SR (2017). Weight loss and improvement in comorbidity: differences at 5%, 10%, 15%, and over. Curr Obes Rep.

[12] Williamson DA, Bray GA, Ryan DH (2015). Is 5% weight loss a satisfactory criterion to define clinically significant weight loss?. Obesity (Silver Spring).

[13] Teixeira PJ, Going SB, Sardinha LB, Lohman TG (2005). A review of psychosocial pre-treatment predictors of weight control. Obes Rev.

[14] Carraça Eliana V, Santos I, Mata J, Teixeira PJ (2018). Psychosocial pretreatment predictors of weight control: a systematic review update. Obes Facts.

[15] Pham Q, Graham G, Carrion C, Morita PP, Seto E, Stinson JN, Cafazzo JA (2019). A library of analytic indicators to evaluate effective engagement with consumer mHealth apps for chronic conditions: scoping review. JMIR Mhealth Uhealth.

[16] Perski O, Blandford A, West R, Michie S (2017). Conceptualising engagement with digital behaviour change interventions: a systematic review using principles from critical interpretive synthesis. Transl Behav Med.

[17] Smith N, Liu S (2020). A systematic review of the dose-response relationship between usage and outcomes of online physical activity weight-loss interventions. Internet Interv.

[18] Sepah SC, Jiang L, Ellis RJ, McDermott K, Peters AL (2017). Engagement and outcomes in a digital Diabetes Prevention Program: 3-year update. BMJ Open Diabetes Res Care.

[19] Painter SL, Ahmed R, Hill JO, Kushner RF, Lindquist R, Brunning S, Margulies A (2017). What matters in weight loss? An in-depth analysis of self-monitoring. J Med Internet Res.

[20] Michaelides A, Major J, Pienkosz Edmund, Wood M, Kim Y, Toro-Ramos T (2018). Usefulness of a novel mobile diabetes prevention program delivery platform with human coaching: 65-week observational follow-up. JMIR Mhealth Uhealth.

[21] Lin P, Grambow S, Intille S, Gallis JA, Lazenka T, Bosworth H, Voils CL, Bennett GG, Batch B, Allen J, Corsino L, Tyson C, Svetkey L (2018). The association between engagement and weight loss through personal coaching and cell phone interventions in young adults: randomized controlled trial. JMIR Mhealth Uhealth.

[22] Spaulding EM, Marvel FA, Piasecki RJ, Martin SS, Allen JK (2021). User engagement with smartphone apps and cardiovascular disease risk factor outcomes: systematic review. JMIR Cardio.

[23] Eysenbach G (2005). The law of attrition. J Med Internet Res.

[24] Castelnuovo G, Pietrabissa G, Manzoni GM, Cattivelli R, Rossi A, Novelli M, Varallo G, Molinari E (2017). Cognitive behavioral therapy to aid weight loss in obese patients: current perspectives. PRBM.

[25] Lawlor E, Islam N, Bates S, Griffin Simon J, Hill Andrew J, Hughes Carly A, Sharp Stephen J, Ahern Amy L (2020). Third-wave cognitive behaviour therapies for weight management: A systematic review and network meta-analysis. Obes Rev.

[26] Armstrong MJ, Mottershead TA, Ronksley PE, Sigal RJ, Campbell TS, Hemmelgarn BR (2011). Motivational interviewing to improve weight loss in overweight and/or obese patients: a systematic review and meta-analysis of randomized controlled trials. Obes Rev.

[27] Michie S, Richardson M, Johnston M, Abraham C, Francis J, Hardeman W, Eccles MP, Cane J, Wood CE (2013). The behavior change technique taxonomy (v1) of 93 hierarchically clustered techniques: building an international consensus for the reporting of behavior change interventions. Ann Behav Med.

[28] Wing RR, Hill JO (2001). Successful weight loss maintenance. Annu Rev Nutr.

[29] American College of Cardiology/American Heart Association Task Force on Practice Guidelines‚ Obesity Expert Panel‚ 2013 (2014). Expert panel report: guidelines (2013) for the management of overweight and obesity in adults. Obesity (Silver Spring).

[30] Postrach E, Aspalter R, Elbelt U, Koller M, Longin R, Schulzke J, Valentini L (2013). Determinants of successful weight loss after using a commercial web-based weight reduction program for six months: cohort study. J Med Internet Res.

[31] DeLuca L, Toro-Ramos T, Michaelides A, Seng E, Swencionis C (2020). Relationship between age and weight loss in Noom: quasi-experimental study. JMIR Diabetes.

[32] Szinay D, Jones A, Chadborn T, Brown J, Naughton F (2020). Influences on the uptake of and engagement with health and well-being smartphone apps: systematic review. J Med Internet Res.

[33] Mitchell ES, Yang Q, Behr H, Ho A, DeLuca L, May CN, Michaelides A (2021). Psychosocial characteristics by weight loss and engagement in a digital intervention supporting self-management of weight. Int J Environ Res Public Health.

[34] Butryn ML, Godfrey KM, Martinelli MK, Roberts SR, Forman EM, Zhang F (2020). Digital self-monitoring: Does adherence or association with outcomes differ by self-monitoring target?. Obes Sci Pract.

[35] Ainsworth B, Steele M, Stuart B, Joseph J, Miller S, Morrison L, Little P, Yardley L (2017). Using an analysis of behavior change to inform effective digital intervention design: how did the PRIMIT website change hand hygiene behavior across 8993 users?. Ann Behav Med.

[36] Thomas DM, Kyle TK, Stanford FC (2015). The gap between expectations and reality of exercise-induced weight loss is associated with discouragement. Prev Med.

[37] Ross KM, Qiu P, You L, Wing RR (2019). Week-to-week predictors of weight loss and regain. Health Psychol.

