# Supplementary material for: The Relationship Between Weight Loss Outcomes and Engagement in a Mobile Behavioral Change Intervention: Retrospective Analysis
Source: JMIR Mhealth Uhealth. 2021 Nov 8;9(11):e30622. doi: 10.2196/30622 (PMC8663454; doi:10.2196/30622)
Supplement: Multimedia Appendix 1 [file mhealth_v9i11e30622_app1.docx]

Multimedia Appendix

Table S1. Summary of MANOVA Results for Weight Change Groups at Each Timepoint

|  | Effect | Wilk’s *Λ* | *F* | df | *p* | Partial η^2^ |
| --- | --- | --- | --- | --- | --- | --- |
| Week 9-16 | | | | | | |
|  | Group^a^ | 0.79 | 197.43 | 2 | <0.001 | 0.17 |
| Week 17-32 | | | | | | |
|  | Group^a^ | 0.80 | 153.50 | 2 | <0.001 | 0.15 |
| Week 33-52 | | | | | | |
|  | Group^a^ | 0.82 | 44.26 | 2 | <0.001 | 0.09 |

^a^ Group variables represent the three weight change groups: Stable (±1%), moderate loss (lost ≥5% to <10%), and high loss (lost ≥10%).

Table S2. Summary of Engagement Measures for Weight Change Groups Across Timepoints.

| **Engagement measures and time** | Weight change categories | | | | | |
| --- | --- | --- | --- | --- | --- | --- |
|  | **Stable (**±1**%)** | | **Moderate loss (5-10%)** | | **High loss (10%)** | |
|  | n | Mean (SD), per week | n | Mean (SD), per week | n | Mean (SD), per week |
| **Days with at least one meal logged** |  |  |  |  |  |  |
| 9-16 weeks | 2594 | 0.88 (0.46) | 6440 | 1.09 (0.45) | 2218 | 1.14 (0.43) |
| 17-32 weeks | 1907 | 0.84 (0.47) | 4369 | 1.1 (0.45) | 2686 | 1.18 (0.42) |
| 33-52 weeks | 525 | 0.59 (0.39) | 1214 | 0.84 (0.39) | 1144 | 0.97 (0.38) |
| Articles read |  |  |  |  |  |  |
| 9-16 weeks | 2594 | 2.87 (1.48) | 6440 | 3.37 (1.49) | 2218 | 3.52 (1.51) |
| 17-32 weeks | 1907 | 2.74 (1.72) | 4369 | 3.63 (1.79) | 2686 | 3.92 (1.74) |
| 33-52 weeks | 525 | 1.74 (1.24) | 1214 | 2.38 (1.22) | 1144 | 2.76 (1.3) |
| Meals logged |  |  |  |  |  |  |
| 9-16 weeks | 2594 | 17.58 (7.1) | 6440 | 23.49 (6.55) | 2218 | 25.68 (6.21) |
| 17-32 weeks | 1907 | 15.83 (7.17) | 4369 | 21.22 (6.88) | 2686 | 24.22 (6.72) |
| 33-52 weeks | 525 | 14.74 (7.33) | 1214 | 19.48 (7.01) | 1144 | 23.14 (6.91) |
| Coach messages |  |  |  |  |  |  |
| 9-16 weeks | 2594 | 0.48 (0.36) | 6440 | 0.54 (0.38) | 2218 | 0.57 (0.4) |
| 17-32 weeks | 1907 | 0.41 (0.3) | 4369 | 0.47 (0.34) | 2686 | 0.51 (0.38) |
| 33-52 weeks | 525 | 0.26 (0.22) | 1214 | 0.34 (0.26) | 1144 | 0.38 (0.29) |
| Steps |  |  |  |  |  |  |
| 9-16 weeks | 2594 | 28546.94 (19570.15) | 6440 | 31966 (21240.98) | 2218 | 37009.37 (22526.54) |
| 17-32 weeks | 1907 | 28036.59 (19499.63) | 4369 | 31758.03 (20823.1) | 2686 | 37191.38 (23573.03) |
| 33-52 weeks | 525 | 27404.34 (18404.96) | 1214 | 31114.64 (23198.8) | 1144 | 35472.55 (23836.5) |
| Weigh ins |  |  |  |  |  |  |
| 9-16 weeks | 2594 | 0.8 (0.45) | 6440 | 1.03 (0.45) | 2218 | 1.1 (0.42) |
| 17-32 weeks | 1907 | 0.73 (0.43) | 4369 | 1 (0.44) | 2686 | 1.08 (0.41) |
| 33-52 weeks | 525 | 0.49 (0.33) | 1214 | 0.65 (0.32) | 1144 | 0.77 (0.33) |
| Exercises |  |  |  |  |  |  |
| 9-16 weeks | 2594 | 2.35 (2.83) | 6440 | 2.54 (3.02) | 2218 | 2.81 (3.22) |
| 17-32 weeks | 1907 | 2.27 (2.71) | 4369 | 2.5 (2.87) | 2686 | 2.99 (3.27) |
| 33-52 weeks | 525 | 2.5 (2.75) | 1214 | 2.63 (2.89) | 1144 | 3.32 (3.37) |
